# The RIG-I pathway is involved in peripheral T cell lymphopenia in patients with dermatomyositis

**DOI:** 10.1186/s13075-019-1905-z

**Published:** 2019-05-29

**Authors:** Lu Zhang, Qisheng Xia, Wenli Li, Qinglin Peng, Hanbo Yang, Xin Lu, Guochun Wang

**Affiliations:** 1 Department of Rheumatology, Beijing Key Lab for Immune-Mediated Inflammatory Diseases, China-Japan Friendship Hospital, 2 Yinhua Road, Chaoyang District, Beijing, 100029 China; 2 Institute of Clinical Medical Sciences, China-Japan Friendship Hospital, Beijing, 100029 China

**Keywords:** RIG-I, T cell lymphopenia, Apoptosis, Dermatomyositis

## Abstract

**Background:**

Peripheral T cell lymphopenia is a clinical phenomenon in some patients with dermatomyositis (DM). Patients with T cell lymphopenia are more susceptible to life-threatening infections. However, the pathogenesis of T cell lymphopenia remains unclear. In this study, we aimed to determine retinoic acid-inducible gene I (RIG-I) expression in peripheral T lymphocytes and explore the correlation between RIG-I and T cell lymphopenia in DM.

**Methods:**

The mRNA and protein expression levels of RIG-I were determined in peripheral T lymphocytes of 26 treatment-naive DM patients by q-PCR and Western blot. The apoptosis of peripheral T lymphocytes was detected by flow cytometry. The associations between RIG-I expression levels and clinical characteristics were investigated. In Jurkat cell, we examined the relationship between RIG-I and cell apoptosis following RIG-I overexpression or activation by specific ligand (pppRNA). The CRISPR/Cas9 gene editing system was used for RIG-I knockout. Fas and caspase 3 were identified by Western blot. CCK8 colorimeter was performed to monitor cell proliferation.

**Results:**

In DM patients, we observed the peripheral T lymphocyte count decreased notably while the apoptosis of T lymphocytes increased significantly compared with healthy control. RIG-I expression levels in peripheral T cell correlated negatively with T cell count in DM patients. RIG-I protein expression decreased significantly, and the number of T cell increased when disease was improved. In Jurkat cells, increased apoptosis and elevated expression of Fas and cleaved-caspase 3 protein were observed following RIG-I overexpression or RIG-I-specific ligand (pppRNA) activation. Meanwhile, the proliferation of Jurkat cells was markedly reduced. Whereas, neither cell apoptosis nor the cell viability of the RIG-I knockout clones exhibited significant changes following pppRNA activation.

**Conclusion:**

Our study showed for the first time that negative correlation between the increased RIG-I expression in peripheral T lymphocyte and T cell count in some patients with DM. We demonstrated that highly expressed RIG-I played a critical role in inducing apoptosis and inhibiting proliferation of T lymphocyte in vitro. Therefore, RIG-I-mediated apoptosis may be one of the possible mechanisms of T cell lymphopenia in some patients with DM. These findings expand our existing knowledge on the mechanisms of innate immunity in pathogenesis and provide new therapeutic avenues for DM.

**Electronic supplementary material:**

The online version of this article (10.1186/s13075-019-1905-z) contains supplementary material, which is available to authorized users.

## Background

Dermatomyositis (DM) is autoimmune systemic disease with multiple systems involvement. Peripheral lymphopenia, predominantly T cell lymphopenia, is a common clinical phenomenon, notably in the MDA5^+^ subgroup [1]. Lymphopenia can be used to predict poor prognosis and may recover with the improvement of the overall disease activity [2, 3]. Patients with T cell lymphopenia are more susceptible to opportunistic infections and life-threatening infections [4–6]. However, the pathogenesis of T cell lymphopenia remains unclear.

Several studies have shown that innate immunity plays an important role in the pathogenesis of DM [7, 8]. Retinoic acid-inducible gene (RIG)-like receptors (RLRs) are cytoplasmic receptors that can recognize and bind double-stranded viral RNA molecules and in turn activate antiviral innate immunity [9–11]. The RLR family consists of three members, including the retinoic acid-inducible gene I (RIG-I), the melanoma differentiation-associated gene (MDA5) and the 1aboratory of genetics and physiology 2 (LGP2) [12]. In the present study, we analyzed RIG-I expression levels in peripheral T lymphocytes and their correlation with clinical characteristics of patients with DM. Furthermore, we explored the involvement of RIG-I in T cell apoptosis in vitro analysis.

## Materials and methods

### Study population

A total of 26 hospitalized treatment-naive DM patients were recruited from the Department of Rheumatology in the China-Japan Friendship Hospital between September 2015 and September 2016. A clinical diagnosis of DM was based on the classification criteria established by Bohan and Peter [13]. We excluded patients suffering from infectious diseases. Patients who presented with accompanying malignancy, overlap syndromes or infection history of hepatitis virus (HAV, HBV, HCV, HDV, and HEV), and human immunodeficiency virus, were excluded. We also excluded the patients with myeloproliferative disease which were determined by peripheral blood routine and blood smear or bone marrow morphology test when necessary. Meanwhile, pregnant and lactating women were excluded. The study protocol was approved by the Ethics Committee of China-Japan Friendship. Hospital written informed consent was obtained from each participant. The study was conducted according to the Declaration of Helsinki (2000).

### Clinical assessment

The physical examinations and laboratory investigations were conducted for all patients at the time of blood sample collection. The number of peripheral lymphocytes and T lymphocyte obtained from routine blood count and T lymphocyte subgroups by flow cytometry (FCM). We conducted detailed records of clinical information, including age at onset, course of disease (recorded monthly), muscle strength, rash (heliotrope rash, Gottron rash, periungual erythema, and “mechanics hand”), and interstitial lung disease (ILD). Muscle strength and severity of rash were measured using the manual muscle test (MMT8) proposed by the International Myositis Outcome Assessment Collaborative Study (IMACS) (http://www.niehs.nih.gov/research/resources/imacs/diseaseactivity/index.cfm) and the modified Cutaneous Dermatomyositis Disease Area and Severity Index (CDASI) [14]. Clinical diagnosis of ILD was based on impaired lung function and typical high-resolution computed tomography (HRCT) features (widespread ground-glass attenuation, intralobular lines/irregular interlobular septal thickening, and honeycombing).

### Cell culture

Jurkat cell line was a gift of the Cell Resource Center, in the Peking Union Medical College (CRC/PUMC). They were maintained in RPMI-1640 (Gibco, Carlsbad, CA, USA) in the presence of 10% (*v*/*v*) fetal bovine serum (Gibco), 100 U/ml penicillin (Gibco), 100 μg/ml streptomycin (Gibco), and 2 mmol/L l-glutamine (Gibco). The cells were cultured in suspension and maintained at 37 °C in a humidified incubator with 5% CO_2_.

### Apoptosis and cell proliferation assays

The percentage of apoptotic T lymphocyte was detected by flow cytometry(FCM). The assay was based on the detection of labeled annexin V/7-amino-actinomycin D (7-AAD) (BD Bioscience, San Jose, CA, USA). Annexin V^+^/7-AAD^−^ subset and annexin V^+^/7-AAD^+^ subset represented the early and late apoptotic population, respectively. Furthermore, CD95 (BD Bioscience) and Bcl2 (BD Bioscience) were detected as an inducer and an inhibitor of apoptosis, respectively. Bcl2 staining was conducted according to the instructions provided by the manufacturers. Human CD3^+^ T lymphocytes and Jurkat cells were analyzed on a BD FACS Jazz or a FACSAria III flow cytometer using conventional software provided by the manufacturer (BD Bioscience, San Jose, CA, USA). The viable cells were measured with the CCK8 assay (Dojindo Laboratories, Japan) according to the manufacturer’s instructions.

### Isolation of T lymphocytes by magnetically activated cell sorting

The peripheral blood mononuclear cells (PBMCs) were isolated from blood by Ficoll density gradient centrifugation (GE Healthcare, Sweden). Human blood-derived CD3^+^ T lymphocytes were sorted using anti-human CD3 MicroBeads (Miltenyi Biotec, Bergisch Gladbach, Germany) according to the manufacturer’s recommendations.

### RNA isolation and real-time quantitative polymerase chain reaction analysis

Total RNA was extracted from cells using TRIzol (Thermo, Carlsbad, CA, USA). The RNA samples were reverse-transcribed using the TransScript First-Strand cDNA Synthesis SuperMix (TaKaRa, Dalian, China). Quantitative real-time reverse transcriptase PCR was performed using SYBR Green (TaKaRa, Dalian, China) in an ABI 7500 system (Applied Biosystems, Singapore). The PCR conditions were carried out according to the manufacturer’s instructions. Each sample was analyzed in triplicate. The relative levels of the mRNA expression of each gene of interest were normalized based on the mRNA levels of the housekeeping gene GAPDH. The expression levels were calculated according to the formula 2 − ΔCT. The primer sequences for the genes were as follows: human RIG-I, Forward:5′-CTTGGCATGTTACACAGCTGAC-3′, Reverse: 5-TTGGCTTGGGATGTGGTCTAC-3′, human GAPDH, Forward: 5′-GAGAAGGCTGGGGCTCATTTGCA-3′. Reverse:5′-TTGGCCAGGGGTGCTAAGCAGT-3′. Primers were synthesized by Tsingke Biological Technology (Beijing, China).

### Western blotting analysis

Western blotting was performed as described previously [15]. Briefly, protein extracts were isolated from each group of cells using the NP-40 protein lysis buffer, which contained protease inhibitor cocktail (Roche, Germany). Total proteins were separated by 10% or 15% SDS-PAGE and were transferred on polyvinylidene difluoride membranes (Millipore, Bedford, Mass, USA) using a semi-dry Gel Transfer Device (Bio-Rad, Hercules, Calif, USA). The membranes were blocked in 5% non-fat milk and probed with primary antibodies and HRP-conjugated secondary antibodies. Antigen-antibody complexes were visualized using the chemiluminescent ECL (Thermo, USA) detection system and analyzed with a ChemDoc XRS+ image analyzer (Bio-Rad, USA). β-actin was used as an internal control. The intensities of the bands were measured by Image J 1.43U software (NIH Image, Bethesda, MD, USA). The antibodies included anti-RIG-I (Cell Signaling Technology, Danvers, MA, USA), anti-Fas (Abcam, Cambridge, UK), anti-cleaved caspase 3 (Cell Signaling Technology, USA), and anti-β-actin (Sigma, St Louis, MO, USA).

### RIG-I overexpression in Jurkat cells

PUNO1-RIG-I plasmid (Invivogene, San Diego, USA) was transfected into Jurkat cells by Lipofectamine 2000 transfection reagent (Thermo, USA), whereas empty plasmid PUNO1-MCS (Invivogene, San Diego, USA) was used as the control. RIG-I protein expression levels were measured by Western blot analysis. Stable RIG-I overexpressing cells were established by blasticidin selection for 5–7 days.

### pppRNA activation

The RIG-I-specific ligand pppRNA (Invivogene, San Diego, USA) was transfected at a concentration of 0.5 μg/ml using Lipofectamine 2000 transfection reagent (Thermo, USA) and pppRNA control was used as control. The analysis was performed at 12- and 24-h time periods.

### RIG-I gene knockout in Jurkat cells

The CRISPR/Cas9 gene editing system was used for RIG-I knockout in Jurkat cells. The pSpCas9(BB)-2A-GFP (PX458) plasmid was a gift from Feng Zhang (Addgene plasmid # 48138). The experiment was performed as described previously [16]. Briefly, two sgRNAs were designed using Zhang Lab’s CRISPR DESIGN tools (https://zlab.bio/guide-design-resources). The annealed sgRNAs were cloned into the PX458 plasmid and transfected into Jurkat cells by electroporation (Lonza, Germany). The fluorescent cells were sorted by the BD Aria III FACS system (BD, USA). Finally, two clones were obtained and the gene mutations were confirmed by Sanger sequencing (Tsingke Biological Technology) and Western blot analysis. The sgRNAs used were as follows: sgRNA1 site: GGGTCTTCCGGATATAATCC TGG; sgRNA2 site: GGATTATATCCGGAAGACCC TGG. Oligo DNA was synthesized by Tsingke Biological Technology (Beijing, China). The sequencing results are shown in the supplementary data.

### Statistical analysis

The analysis was performed using PASW statistics 18. The comparisons between groups were performed using independent tests for continuous variables and the Pearson chi-square test for categorical variables. The paired *t* test was applied in the follow-up study. Mann-Whitney tests were applied for data of abnormal distribution. For independent samples(*K* ≥ 3), we used the Kruskal-Wallis *H* test and adjust *p* value for comparison between two groups with Bonferroni method. A correlation analysis for the variables of interest was performed using Pearson correlation. Continuous variables were presented as mean ± SD unless otherwise stated. Two-sided values of *p* < 0.05 were considered statistically significant.

## Results

### Baseline characteristics

**Fig. 1:**
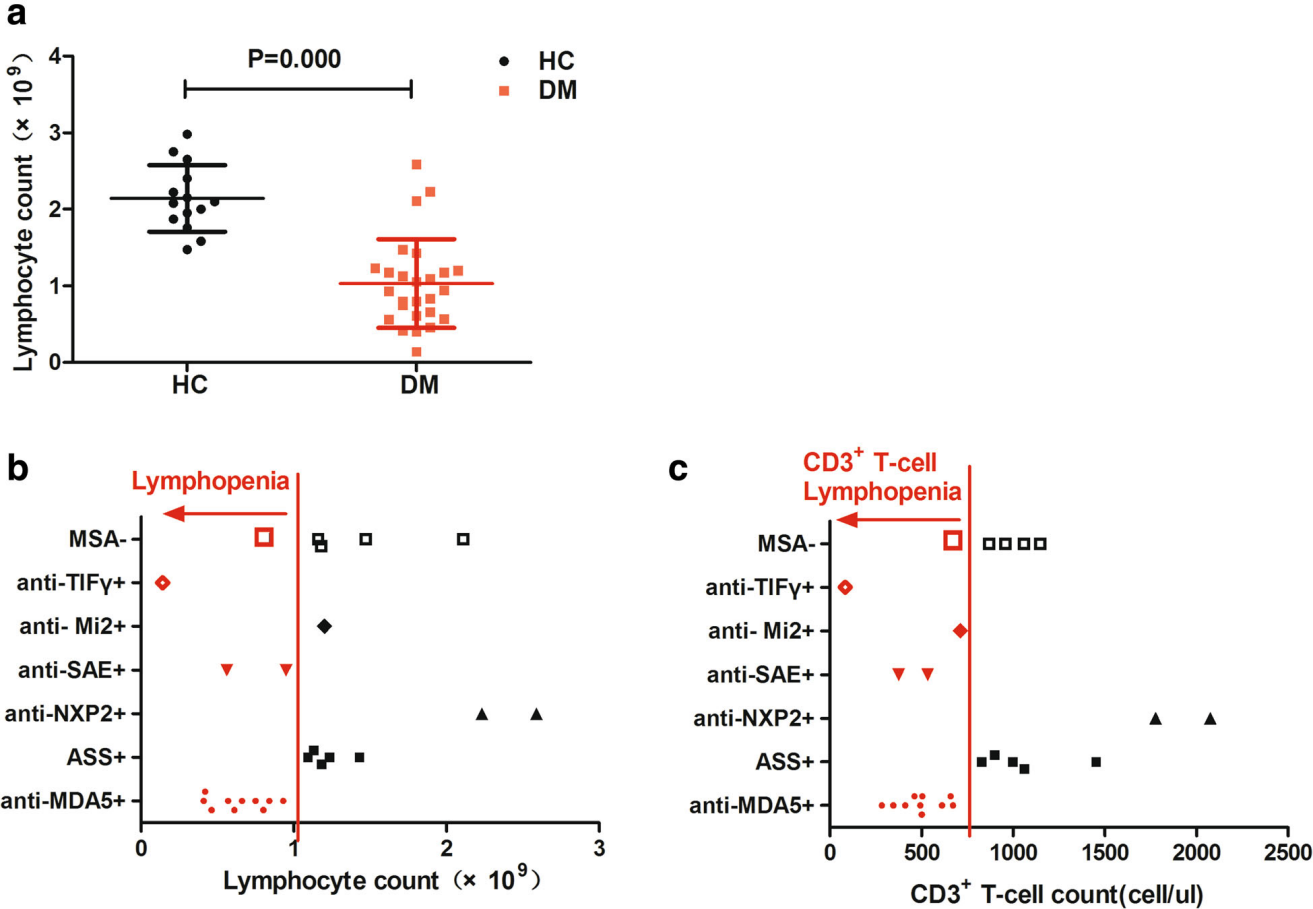
Peripheral lymphocytes count decreased in DM. **a** The average lymphocyte count decreased significantly in DM compared with the normal control group. **b**, **c** The distribution of myositis-specific antibody (MSA) in patients with lymphopenia and T cell lymphopenia. ASS: antisynthetase antibody

The age of the patients ranged from 19 to 71 years, with a mean of 53.6 (± 13.7) years. The average disease duration was 7.69 months (± 6.40, range 1–22 months). The patients were matched to 14 healthy control subjects on the basis of age and sex. The age of the control subjects ranged from 20 to 63 years, and the mean was 47.3 years (± 12.9). A total of 21 patients were positive following testing with myositis-specific antibody (MSA). These subjects included 10 patients who tested positive for anti-MDA5, 2 patients for anti-NXP2, 5 patients for antisynthetase (anti-Jo-1, anti-PL-7, anti-PL12), 1 patient for anti- Mi2, 2 patients for anti-SAE, and 1 patient for anti-TIF. The average lymphocyte count decreased significantly in DM compared with that in the normal control group (1.03 ± 0.58 × 10^9^ vs 2.14 ± 0.43 × 10^9^, *p* = 0.000) (Fig. 1a). Lymphopenia (0.64 ± 0.23 × 10^9^) was noted in 14 patients. The 10 patients who were positive for anti-MDA5 exhibited different degrees of lymphopenia. The remaining 4 patients included 2 positive patients for anti-SAE, 1 positive patient for anti-TIFγ, and 1 negative patient for MSA (Fig. 1b). Meanwhile, T lymphocyte decreased in all 14 patients above (472 ± 165 cell/ul) (Fig. 1c).

### The apoptosis levels of peripheral T cells from DM patients were increased

**Fig. 2:**
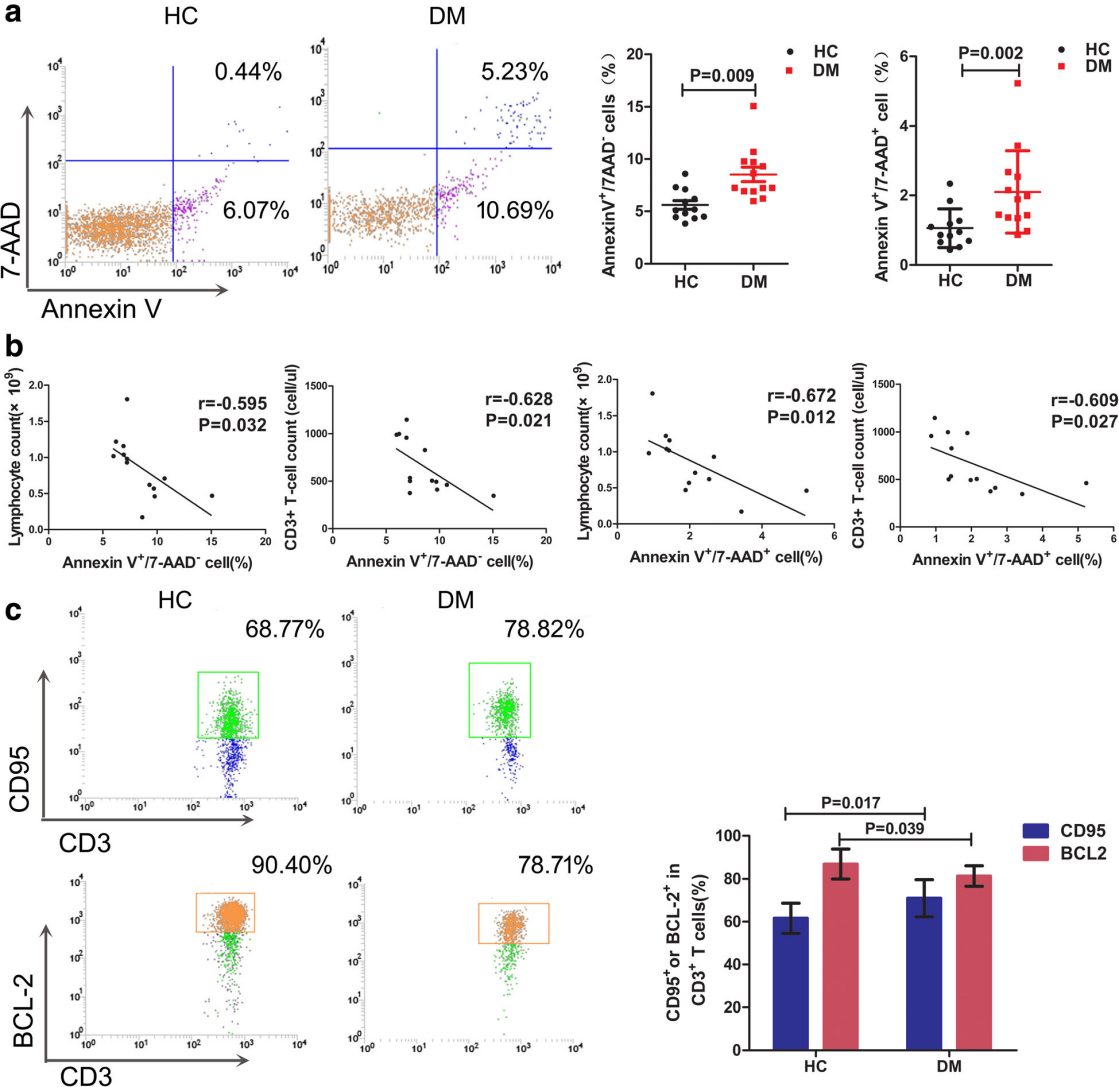
Apoptosis of peripheral lymphocytes detected by flow cytometry.  **a** The proportion of annexin V^+^/7-AAD^−^CD3^+^ cells and annexin V^+^/7-AAD^+^CD3^+^ cells increased significantly compared with that of the healthy control subjects. Representative graphs were shown. **b** The percentage of annexin V^+^/7AAD^−^CD3^+^ cells correlated negatively to peripheral lymphocyte count and T cell count in DM patients. Similarly, the percentage of annexin V^+^/7AAD^+^CD3^+^ cells correlated negatively to peripheral lymphocyte count and T cell count in DM patients. **c** The proportion of CD95^+^ cells increased significantly and the proportion of Bcl2^+^ cells decreased significantly compared with that of the healthy control subjects. The representative graphs were shown

The apoptosis rate of peripheral T cell was detected with the flow cytometer (FCM) in 13 patients and 12 controls. FCM results indicated that the ratio of annexin V^+^/7-AAD^−^ subset and annexin V^+^/7-AAD^+^ subset of the DM patients (*n* = 13) increased significantly compared with those of the healthy control subjects (*n* = 12) (*z* = − 2.611, *p* = 0.009; *z* = − 3.046, *p* = 0.002, respectively) (Fig. 2a). The ratio of early apoptosis cell (annexin V^+^/7AAD^−^CD3^+^ cells) correlated negatively to peripheral lymphocyte count(*r* = − 0.595, *p* = 0.032) and T lymphocyte count in DM patients (*r* = − 0.628, *p* = 0.021). Similarly, the percentage of late apoptosis cell (annexin V^+^/7-AAD^+^CD3^+^ cells) correlated negatively to peripheral lymphocyte count (*r* = − 0.672, *p* = 0.012) and T lymphocyte count(*r* = − 0.609, *p* = 0.027) in DM patients (Fig. 2b). In addition, the proportion of CD95^+^ cells increased (*z* = − 2.393, *p* = 0.017), while the proportion of Bcl2^+^ cells decreased significantly (*z* = − 2.067, *p* = 0.039) compared with that of the healthy control subjects (Fig. 2c). The results indicated that peripheral lymphopenia and T cell lymphopenia were associated with increased apoptosis.

In our study, all the anti-MDA5-positive patients (*n* = 10) presented different levels of lymphopenia. Whereas, patients with anti-ASS+ (*n* = 5) showed a normal lymphocyte count. We compared the percentage of annexin V and CD 95 between anti-MDA5+ (*n* = 6), anti-ASS (*n* = 3), and other patients (*n* = 4, including 2 patients for MSA−, 1 patient for anti-SAE+, 1 patient for anti-TIFγ+). Although the statistical results did not show a significant difference due to the small sample size, the percentage of annexin V and CD 95 in patients with MDA5+ showed trends of elevation (Additional file 1: Figure S1).

### RIG-I expression levels in peripheral T lymphocytes increased significantly and correlated negatively with the lymphocyte count

**Fig. 3:**
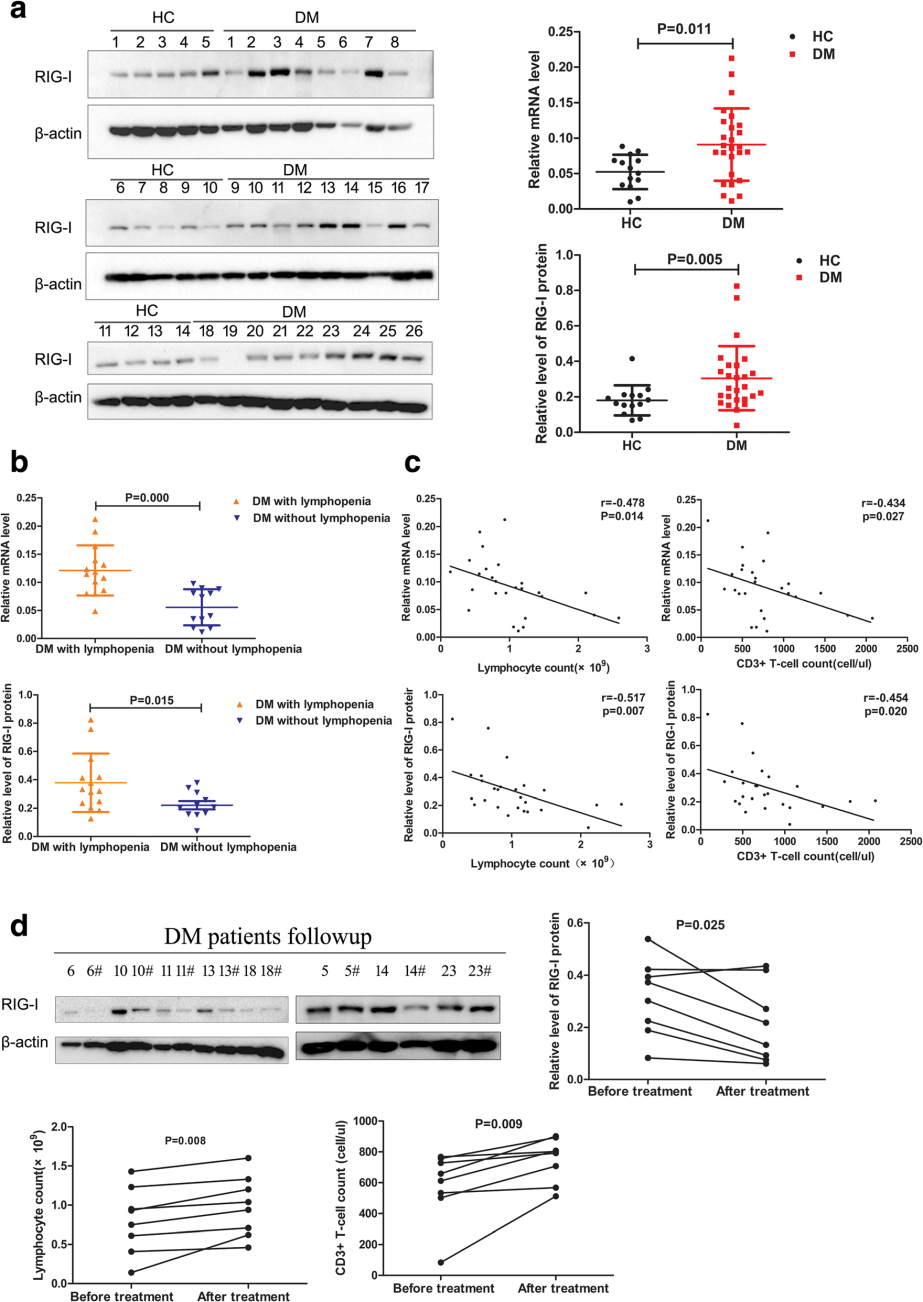
The RIG-I expression level increased significantly in peripheral T lymphocytes and correlated negatively with T lymphocyte count in DM. **a** Western blotting analysis of RIG-I expression in peripheral T lymphocytes of DM patients and healthy controls (HC). DM patients exhibited significantly higher levels of RIG-I mRNA and RIG-I protein. **b** The RIG-I mRNA and protein expression level in T lymphocytes of patients with lymphopenia were significantly higher than those of the patients without lymphopenia. **c** The protein and gene protein expression level of RIG-I correlated negatively with lymphocyte count and T lymphocyte count. **d** The RIG-I expression decreased significantly after therapy. The number of lymphocytes and T lymphocyte were increased at the 3-month follow-up period

DM patients exhibited significantly higher levels of RIG-I mRNA (0.091 ± 0.051 vs. 0.052 ± 0.024, *p* = 0.011) and RIG-I protein (0.30 ± 0.18 vs. 0.18 ± 0.08, *p* = 0.005) in peripheral T lymphocytes than those in healthy control subjects (Fig. 3a). Following subgroup according to the presence of lymphopenia in the patients analysis showed that the mRNA and protein levels of RIG-I in T lymphocytes in patients with lymphopenia were significantly higher than those of patients without lymphopenia (0.121 ± 0.045 vs. 0.056 ± 0.032, *p* = 0.000; 0.379 ± 0.206 vs. 0.213 ± 0.084, *p* = 0.015) (Fig. 3b).

As for the characteristic of high heterogeneity in DM, we compared the expression of RIG-I between anti-MDA5+ (*n* = 10), anti-ASS+ (*n* = 5), and the remaining patients (*n* = 11, including 5 patients for MSA−, 2 patients for anti-NXP2+, 2 patients for anti-SAE+, 1 patient for anti-TIFγ+, 1 patient for anti-Mi2+). The relative mRNA level of RIG-I significantly increased in patients with MDA5+ compared to patients with ASS+ or other myositis-specific antibodies(*z* = − 2.449, *p* = 0.042; *z* = − 2.887, *p* = 0.012, respectively). Similar with the apoptosis markers, relative levels of RIG-I protein also showed increasing trends without a statistical difference (Additional file 2: Figure.S2).

A negative correlation between the gene and protein expression levels of RIG-I and the lymphocyte count was observed (*r* = − 0.478, *p* = 0.014; *r* = − 0.517, *p* = 0.007) (Fig. 3c). Similarly, there was a negative correlation between the gene and protein expression level of RIG-I and peripheral T lymphocyte count (*r* = − 0.434, *p* = 0.027; *r* = − 0.454, *p* = 0.020) (Fig. 3c). Furthermore, there was a positive correlation between gene and protein expression levels of RIG-I and the CDASI score (*r* = 0.455, *p* = 0.02; *r* = 0.507, *p* = 0.008) (Additional file 3: Figure S3A). The patients with ILD exhibited higher RIG-I gene and protein expression levels than those without lung disease (0.112 ± 0.048 vs. 0.051 ± 0.029; *p* = 0.002, 0.363 ± 0.190 vs. 0.187 ± 0.078, *p* = 0.014) (Additional file 3: Figure S3B). However, RIG-I levels indicated no correlation with serum creatine kinase (CK) levels (*p* = 0.356) and the MMT8 score (*p* = 0.284).

A total of 8 patients were followed up after 3 months of therapy. All patients were more or less improved notably with regard to their rash and symptoms and their muscle strength. The RIG-I expression levels significantly decreased (0.316 ± 0.146 vs. 0.213 ± 0.151, *p* = 0.025). The number of lymphocytes (0.81 ± 0.42 vs. 0.99 ± 0.39, *p* = 0.008) and T lymphocytes (580.5 ± 223.6 vs. 748.3 ± 143.0, *p* = 0.009) were increased at the 3-month follow-up period (Fig. 3d).

### RIG-I overexpression and pppRNA activation induced apoptosis and reduced the viability of Jurkat cells

**Fig. 4:**
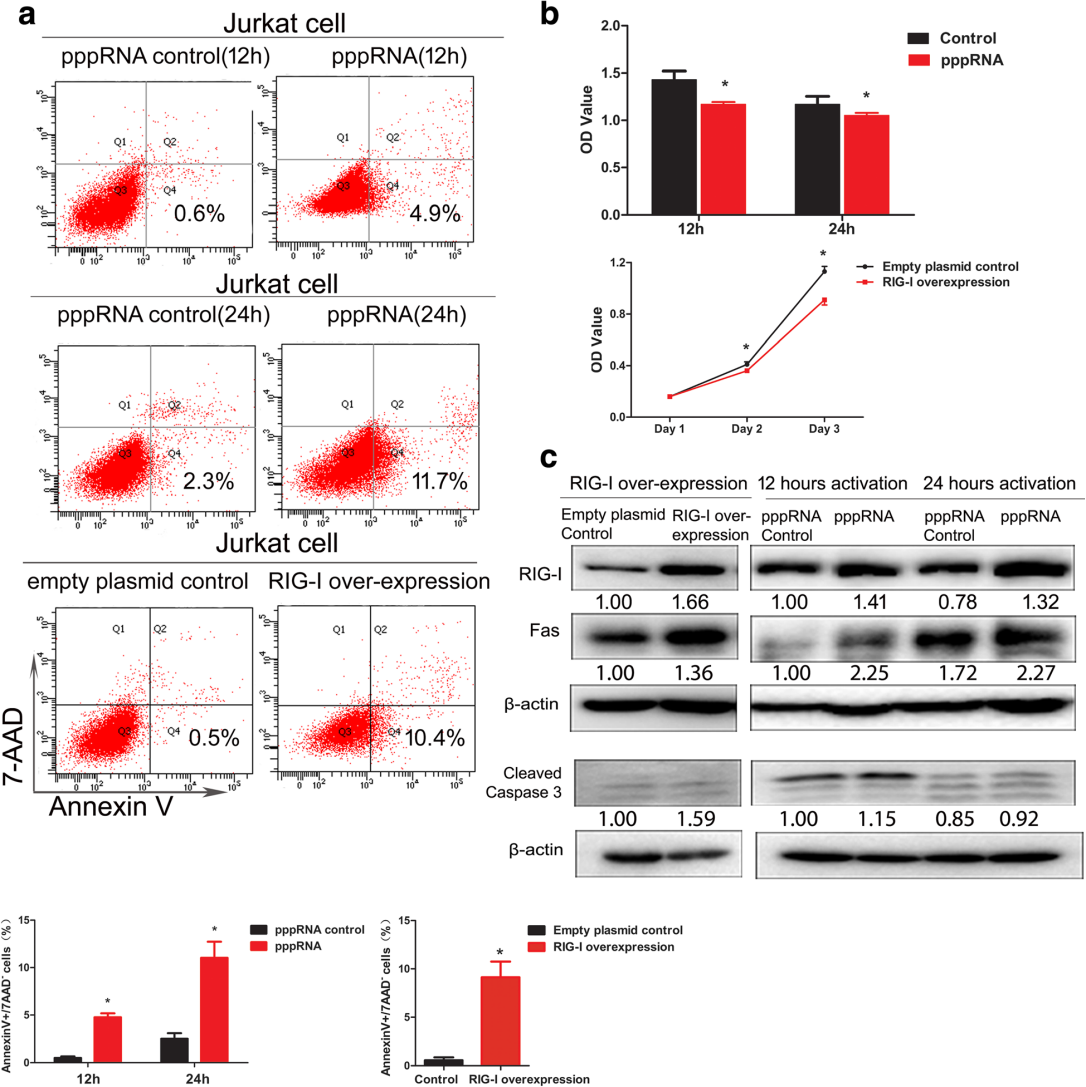
RIG-I overexpression and pppRNA activation induced apoptosis and reduced the viability of Jurkat cells. **a** A significant increase of apoptotic populations (annexin V^+^/7-AAD^−^) was observed with pppRNA 12 h and 24 h activation or RIG-I overexpression. **b** The proliferation of Jurkat cells was inhibited significantly after pppRNA 12 h and 24 h activation or RIG-I overexpression. **c** RIG-I protein expression was upregulated in Jurkat cell and the elevated expressions of Fas and caspase 3 increased after pppRNA 12 h and 24 h activation or RIG-I overexpression. The relative intensity values were measured with ImageJ software, and the results are shown below the blot. The Western blot analysis was performed at least two times, and representative results are shown. *p ≤ 0.05

RIG-I protein expression levels were upregulated in Jurkat cells following plasmid PUNO1-RIG-I transfection or pppRNA activation. A significant increase of the early apoptotic populations (annexin V^+^/7-AAD^−^) was observed following RIG-I overexpression (9.13 ± 1.63 vs. 0.57 ± 0.31, *p* = 0.001) and pppRNA 12-h and 24-h activation (4.07 ± 0.21 vs. 0.53 ± 0.15; *p* = 0.000, 11.03 ± 1.70 vs. 2.53 ± 0.59; *p* = 0.001) (Fig. 4a). However, no significant difference of the late apoptotic subset (annexin V^+^/7-AAD^+^) was found after RIG-I overexpression and stimulation of pppRNA (data not shown). The proliferation of Jurkat cells was inhibited significantly following RIG-I overexpression compared with those in the control group (1.13 ± 0.44 vs. 0.91 ± 0.45, *p* = 0.000) (Fig. 4b). Activation of RIG-I by pppRNA caused a marked reduction in cell viability within 12 and 24 h (1.43 ± 0.89 vs. 1.17 ± 0.25; *p* = 0.000, 1.17 ± 0.82 vs. 1.05 ± 0.27; *p* = 0.017) (Fig. 4b). Furthermore, the elevated expressions levels of Fas and cleaved caspase 3 indicated the induction of apoptosis (Fig. 4c). These results indicated a high sensitivity of Jurkat cells with regard to the induction of apoptosis by RIG-I overexpression or RIG-I agonists.

### RIG-I is required in pppRNA-stimulated apoptosis in Jurkat cells

**Fig. 5:**
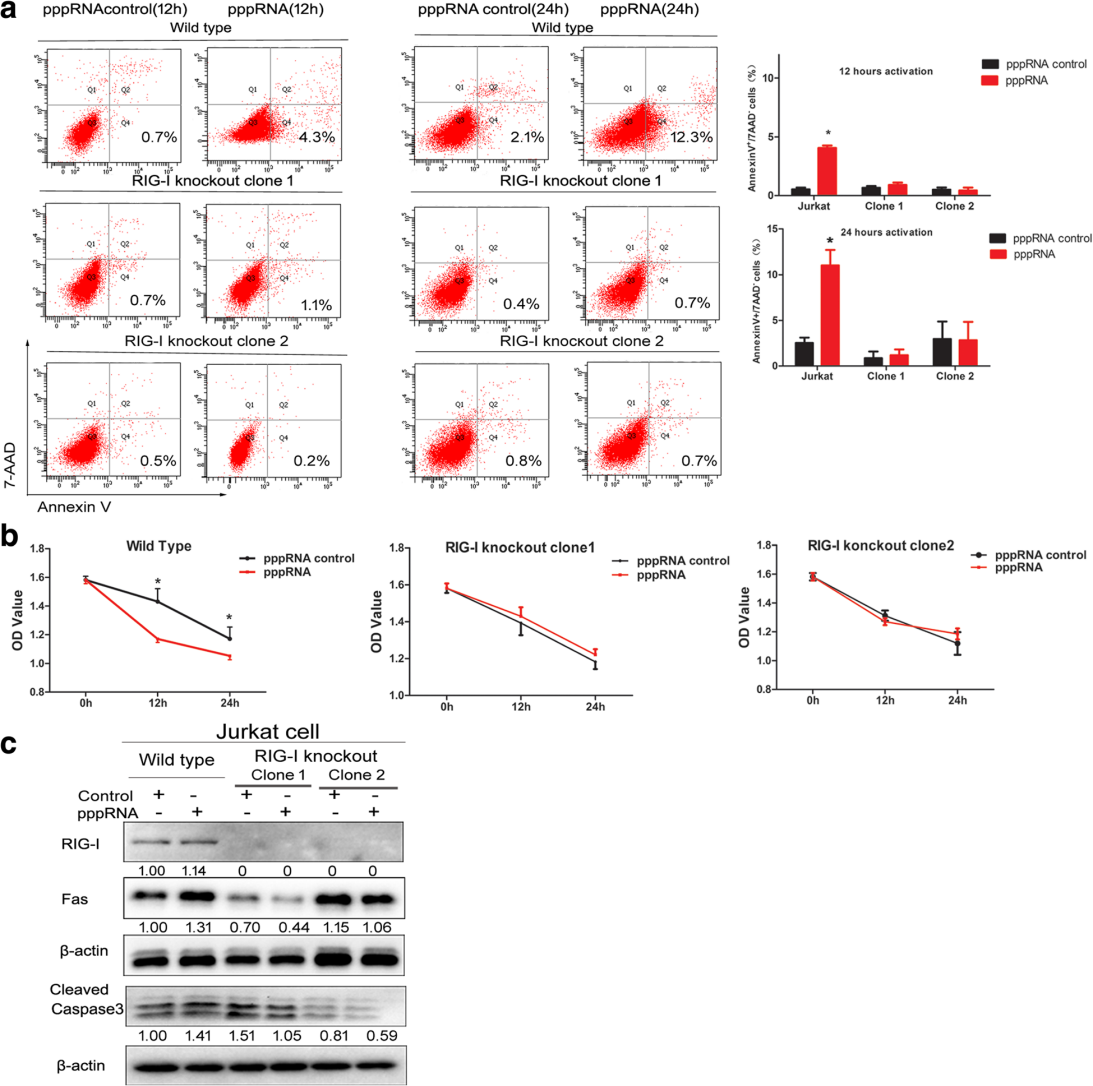
Knockout of RIG-I inhibited pppRNA-stimulated apoptosis in Jurkat cells. **a** No obvious change in apoptotic populations (annexin V^+^/7-AAD^−^) could be seen by flow cytometry in RIG-I knockout clones after pppRNA activation. **b** No obvious changes appear on the proliferation of RIG-I knockout clones after pppRNA activation. **c** The loss expression of RIG-I in two RIG-I knockout clones. The expression of Fas and caspase 3 did not increase after pppRNA activation. The relative intensity values were measured with ImageJ software, and the results are shown below the blot. The Western blot analysis was performed at least two times, and representative results are shown. *p ≤ 0.05

In order to verify the role of RIG-I in pppRNA-stimulated apoptosis, we constructed RIG-I knockout cell lines by the CRISPR/Cas9 editing system. Two RIG-I knockout clones were obtained successfully which were confirmed by gene sequencing (Additional file 4: Figure S4) and loss expression of RIG-I in Western blot (Fig. 5c). No change in the ratio of annexin V^+^/7-AAD^−^ populations could be seen in RIG-I knockout clones after pppRNA 12 and 24 h activation (Fig. 5a). Similarly, no changes were evident with regard to the proliferation of the RIG-I knockout clones following pppRNA activation (Fig. 5b). In addition, the expression levels of Fas and cleaved caspase 3 did not increase following pppRNA activation (Fig. 5c).

## Discussion

To the best of our knowledge, the present study represents the first report indicating that RIG-I expression is significantly increased in peripheral T lymphocytes from DM patients compared with those of healthy control subjects. The RIG-I expression levels correlated positively with the CDASI score. The patients with ILD exhibited higher RIG-1 gene expression levels than those without lung disease. RIG-I expression levels in T lymphocytes were significantly decreased following therapy.

Although peripheral T cell lymphopenia present in some treatment-naive DM patients, the mechanism is far from clear. It is important to note that a significant negative correlation was observed between RIG-I expression levels and T lymphocyte count. Based on the inverse correlation between RIG-I and the T lymphocyte count, the mechanism of RIG-I in T cell lymphopenia was further explored. Previous studies indicated that targeted activation of RIG-I by pppRNA induced apoptosis in human melanoma, ovarian cancer cells, and cervical cancer cells [17–19]. However, whether the activation of RIG-I can further increase the apoptosis of T lymphocytes has not been investigated. Concomitantly, the relationship between apoptosis and T-cell lymphopenia was explored in the present study.

We detected a significant increase of early and late apoptotic populations (annexin V^+^/7-AAD^−^ and annexin V^+^/7-AAD^+^) in peripheral T cells from DM patients by FCM compared with those of the normal subjects. Furthermore, the apoptosis promoter CD95 was upregulated, whereas the anti-apoptotic protein Bcl2 was downregulated in T lymphocyte of DM patients. The proportion of annexin V^+^7AAD^−^CD3^+^ T cells and annexin V^+^7AAD^+^CD3^+^ T cells indicated negative correlations with the lymphocyte count and T lymphocyte count in DM patients. The data suggested that peripheral lymphopenia and T cell lymphopenia were associated with increased apoptosis in DM.

To clarify the relationship between RIG-I and apoptosis of T lymphocytes, RIG-I was overexpressed by a PUNO1-RIG-I plasmid and activated by the specific ligand pppRNA in Jurkat cells. Results indicated that both RIG-I overexpression and stimulation of pppRNA in Jurkat cells could increase early apoptotic populations (annexin V^+^/7-AAD^−^) and reduce cell viability. However, the late apoptotic subset (annexin V^+^/7-AAD^+^) did not show a significant difference. The possible explanation is that the stimulation time and harvesting time may affect the proportion of late apoptosis cells. Western blotting verified the elevated expression levels of Fas and cleaved caspase 3. Furthermore, the RIG-I gene was targeted with CRISPR/Cas9 interference. As a result, neither cell apoptosis nor the cell viability of the RIG-I knockout clones exhibited significant changes following pppRNA activation. Also, the Fas and cleaved caspase 3 protein did not increase in RIG-I knockout Jurkat cell clones following pppRNA activation. The aforementioned results indicated that RIG-I played a critical role in pppRNA-induced apoptosis of Jurkat cells.

The present study reported for the first time that RIG-I overexpression and activation by pppRNA could induce apoptosis in T lymphocyte lines. Therefore, RIG-I-mediated apoptosis may be one of the possible mechanisms of T cell lymphopenia in some patients with DM. Type I interferon-mediated innate immune mechanism plays an important role in the pathogenesis of dermatomyositis [8, 20–22]. RIG-I is a member of the family of RIG-like receptors (RLRs) that primarily bind to 5′-phosphorylated regions of single RNA and long double-stranded RNA molecules. Yoneyama demonstrated that RIG-I could act as a no-Toll-like pattern recognition receptor that recognizes and binds cytoplasmic viral RNA, which results in a cascade of events leading to increased transcription of type I interferon [23]. Recent studies have shown that RIG-I is significantly upregulated in the skeletal muscle of dermatomyositis patients [24, 25]. Stimulation of human myotubes with a ligand of RIG-I produced a significant secretion of interferon-β and the upregulation of class I MHC [24]. Therefore, the present study provided additional evidence for the involvement of the type I interferon pathway in DM pathogenesis and indicated its distinct roles in the functions of peripheral blood and muscle cells.

Although decreased peripheral T cells are often observed in DM patients with anti-MDA5+, T cell lymphopenia and upregulated expression of RIG-I were also observed in 5 patients without anti-MDA5 antibody (2 patients for anti-SAE+, 1 patient for anti-TIFγ+ 2, 1 patient with Mi2+, and 1 patient for MSA−) in our study. Our study focuses on the RIG-I pathway in T cell lymphopenia. Furthermore, experiments in vitro demonstrated that RIG-I could trigger apoptosis and reduce cell proliferation of lymphocytes. Therefore, we suggested that the RIG-I pathway may be one of the paths which involved in lymphopenia in some DM patients.

Future studies can elucidate the upstream and downstream mediators of the molecular pathway of RIG-I. This will contribute to a further understanding of the pathological mechanisms of DM. One recent study suggested that lnc-Lsm3b, an IFN-inducible long noncoding RNA located on chromosome 6, could compete with viral RNAs in the binding of RIG-I monomers. Lnc-Lsm3b could also feedback inactivate the RIG-I innate function at the late stages of the innate immune response. The deficiency of lnc-Lsm3b notably enhances RIG-I-initiated IFN production [26]. However, the experiment only based on a mouse model, the corresponding transcript of lnc-Lsm3b in human is not identified. Therefore, lnc-Lsm3b, as a hypothesized upstream pathway of RIG-I, whether involved in the upregulated expression of RIG-I in DM, requires further investigation. In addition, type I interferon is a downstream molecular mediator of RIG-I. The study conducted by Besch. suggested that the induction of apoptosis by RIG-I was independent of type I interferon in human melanoma cells [17]. Additional studies are required to clarify whether type I interferon is involved in the RIG-I-mediated apoptosis of T lymphocyte lines.

We acknowledge the limitation of this study. First, as extremely useful tools for in vitro study, Jurkat cells were used to explore the association between RIG-I and apoptosis instead of human T lymphocytes. On this basis, further in-depth studies with freshly isolated T lymphocytes from DM patients maybe more helpful to verify the correlation between RIG-I and T cell lymphopenia in DM. Furthermore, the mechanism of T cell apoptosis induced by RIG-I is far from clear. We only detected the elevated expressions level of Fas after RIG-I overexpression or RIG-I agonists. However, whether RIG-I directly regulates the Fas/Fas ligand pathway to induce apoptosis needed to be further explored. Finally, RIG-I and MDA5 are of the same family (RLRs) and both intracellular receptors for detecting RNA viruses. And clinically, T cell lymphopenia is distinctly seen in the MDA5+ subgroup. Previous studies have suggested activation MDA-5 poly(I:C) strongly induced apoptosis in human melanoma cells. It is reasonable to presume that MDA5 pathway may also play an important role in the progression of lymphopenia in DM patients. However, we did not detect the expression of MDA5 and explore the correlation between MDA5 pathway and lymphopenia in our research. Our study just shows that RIG-I pathway is one of the paths which involved in lymphopenia. Whether there are some interactions between the RIG-I and MDA5 pathway in lymphopenia needs to be further studied.

In summary, our study clarified that RIG-I was significantly increased in peripheral T lymphocytes of some DM patients. Functional experiments indicated that RIG-I could trigger apoptosis and reduce cell proliferation of lymphocytes. Therefore, the RIG-I pathway may play a significant role in the progression of T cell lymphopenia in some DM patients. These findings expand our existing knowledge on the mechanisms of innate immunity and provide new therapeutic avenues for targeting DM.

## Additional files

Additional file 1: **Figure S1.** The percentage of annexin V (A) and CD 95 (B) in patients with MDA5+ compared to patients with ASS+ or other myositis-specific antibodies. (TIF 748 kb)

Additional file 2: **Figure S2**. RIG-I mRNA (A) and protein (B) expression level expression in patients with MDA5+ compared to patients with ASS+ or other myositis-specific antibodies. (TIF 382 kb)

Additional file 3: **Figure S3.** Relationship between RIG-I expression level in peripheral T lymphocytes and clinical characteristics. (A) A positive correlation between the gene and protein expression levels of RIG-I and the CDASI score. (B) The patients with ILD exhibited higher RIG- I gene and protein expression levels than those without ILD. (TIF 521 kb)

Additional file 4: **Figure S4.** Gene mutation information of RIG-I knockout Jurkat cell clones. (A) Illustration of the targeting site of designed guide RNA sequence, the site was 40 bases below the translation start site. (B, C) the sequencing results of Jurkat RIG-I knockout clone1 and clone 2. After the monoclones of Jurkat RIG-I knockout were obtained, the DNA region that contained the targeting site was amplicated. Then, the PCR products were cloned into T vector and sequenced by Sanger sequencing. (TIF 1270 kb)

## Abbreviations

7-AAD: 7-Amino-actinomycin D
ASS: Antisynthetase antibody
CDASI: Cutaneous Dermatomyositis Disease Area and Severity Index
CRC/PUMC: Cell Resource Center in the Peking Union Medical College
DM: Dermatomyositis
FCM: Flow cytometry
HRCT: High-resolution computed tomography
ILD: Interstitial lung disease
IMACS: International Myositis Outcome Assessment Collaborative Study
LGP2: Laboratory of genetics and physiology 2
MDA5: Melanoma differentiation-associated gene
MMT8: Manual muscle test
MSA: Myositis-specific antibody
PBMCs: Peripheral blood mononuclear cells
RIG-I: Retinoic acid-inducible gene I
RT-PCR: Real-time quantitative polymerase chain reaction analysis
