# Supplementary figures and images for: The RIG-I pathway is involved in peripheral T cell lymphopenia in patients with dermatomyositis
Source: Arthritis Res Ther. 2019 May 29;21:131. doi: 10.1186/s13075-019-1905-z (PMC6542107; doi:10.1186/s13075-019-1905-z)

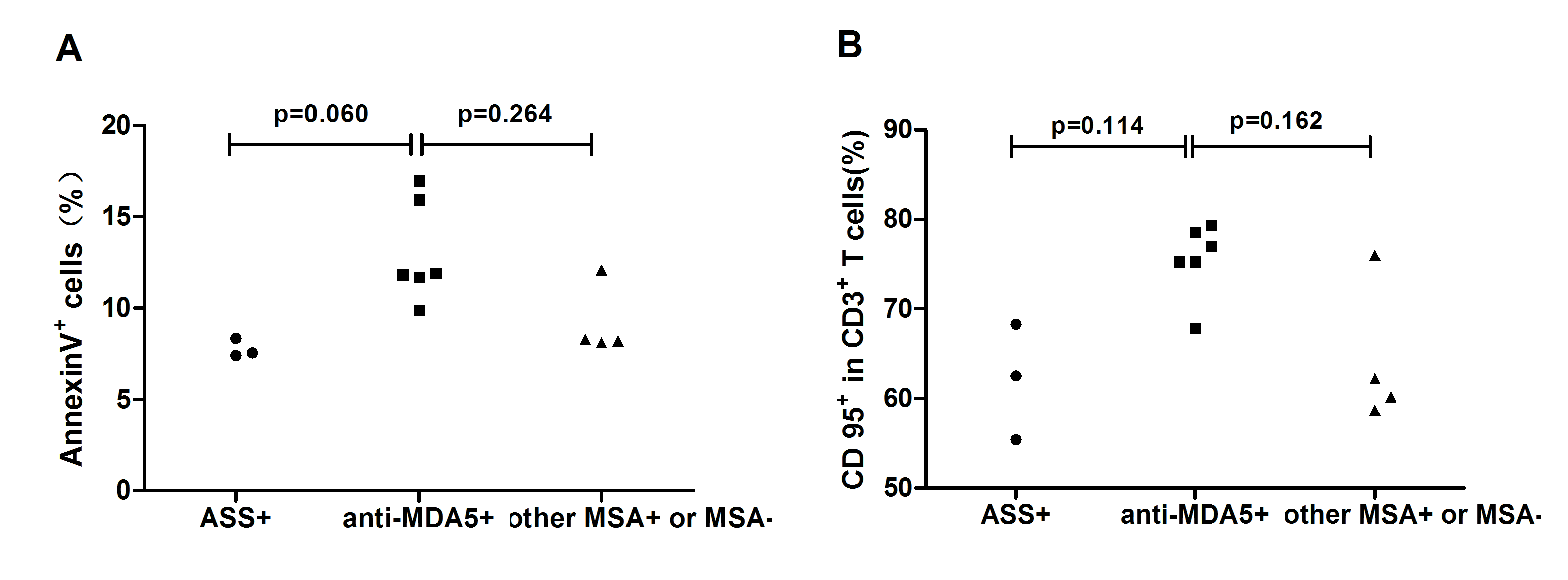

Supplement: Supplementary file 1 — Figure S1. The percentage of annexin V (A) and CD 95 (B) in patients with MDA5+ compared to patients with ASS+ or other myositis-specific antibodies. (TIF 748 kb) [file 13075_2019_1905_MOESM1_ESM.tif]

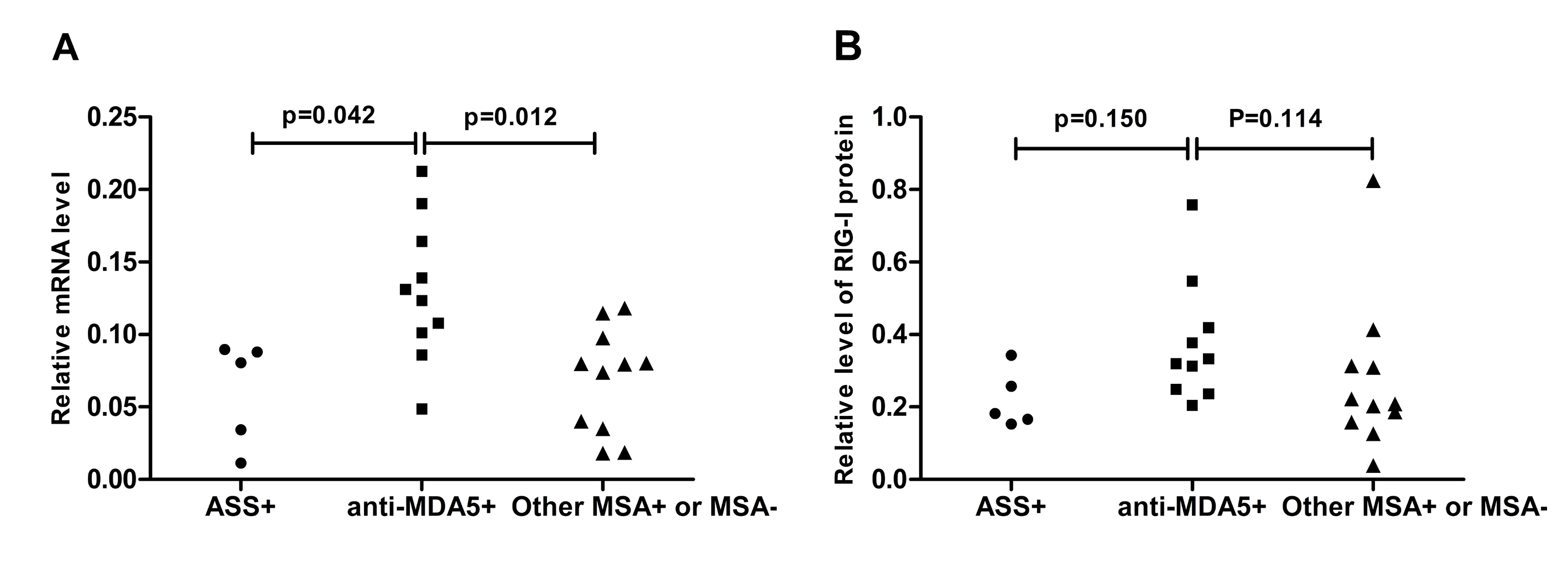

Supplement: Supplementary file 2 — Figure S2. RIG-I mRNA (A) and protein (B) expression level expression in patients with MDA5+ compared to patients with ASS+ or other myositis-specific antibodies. (TIF 382 kb) [file 13075_2019_1905_MOESM2_ESM.tif]

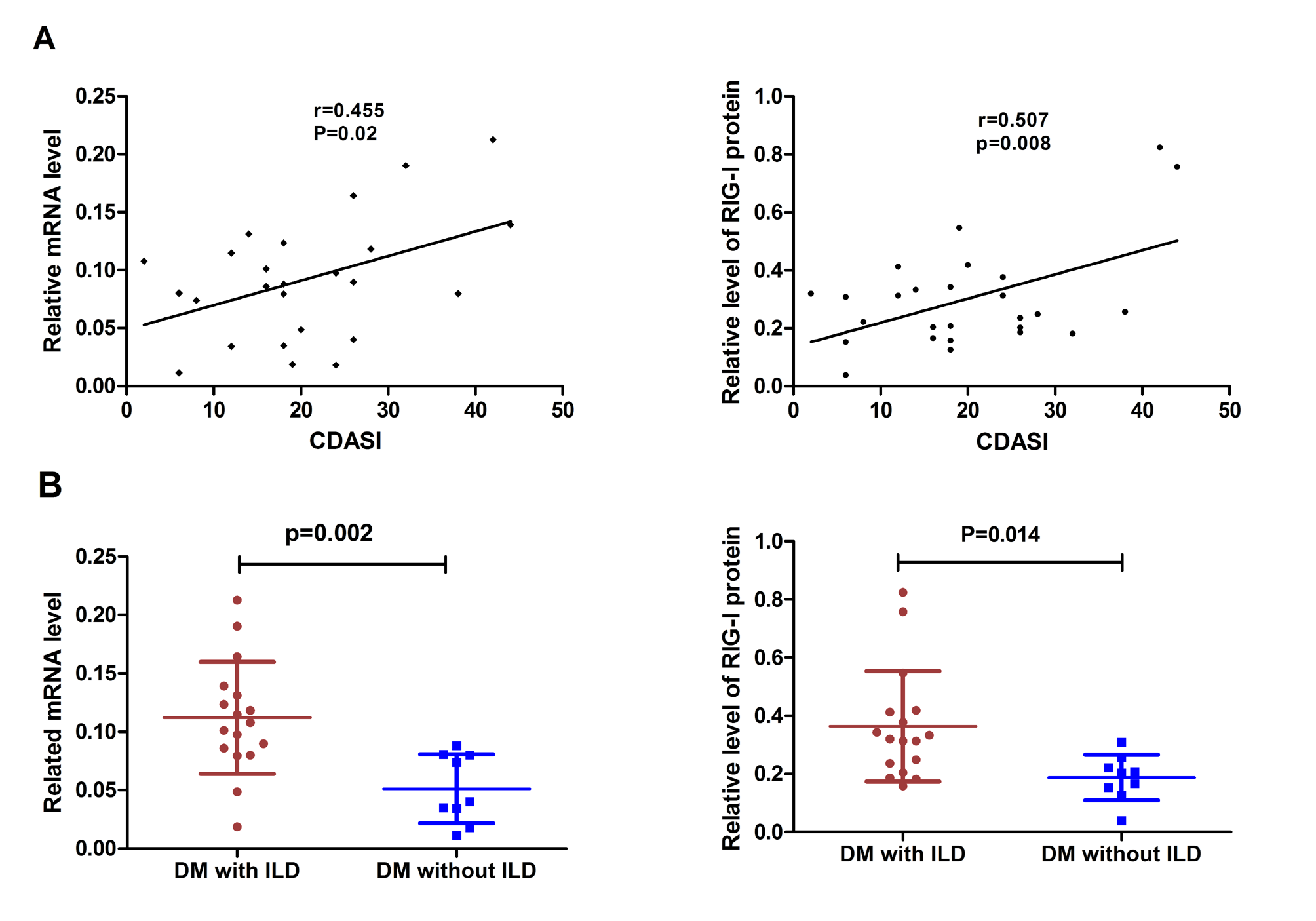

Supplement: Supplementary file 3 — Figure S3. Relationship between RIG-I expression level in peripheral T lymphocytes and clinical characteristics. (A) A positive correlation between the gene and protein expression levels of RIG-I and the CDASI score. (B) The patients with ILD exhibited higher RIG- I gene and protein expression levels than those without ILD. (TIF 521 kb) [file 13075_2019_1905_MOESM3_ESM.tif]

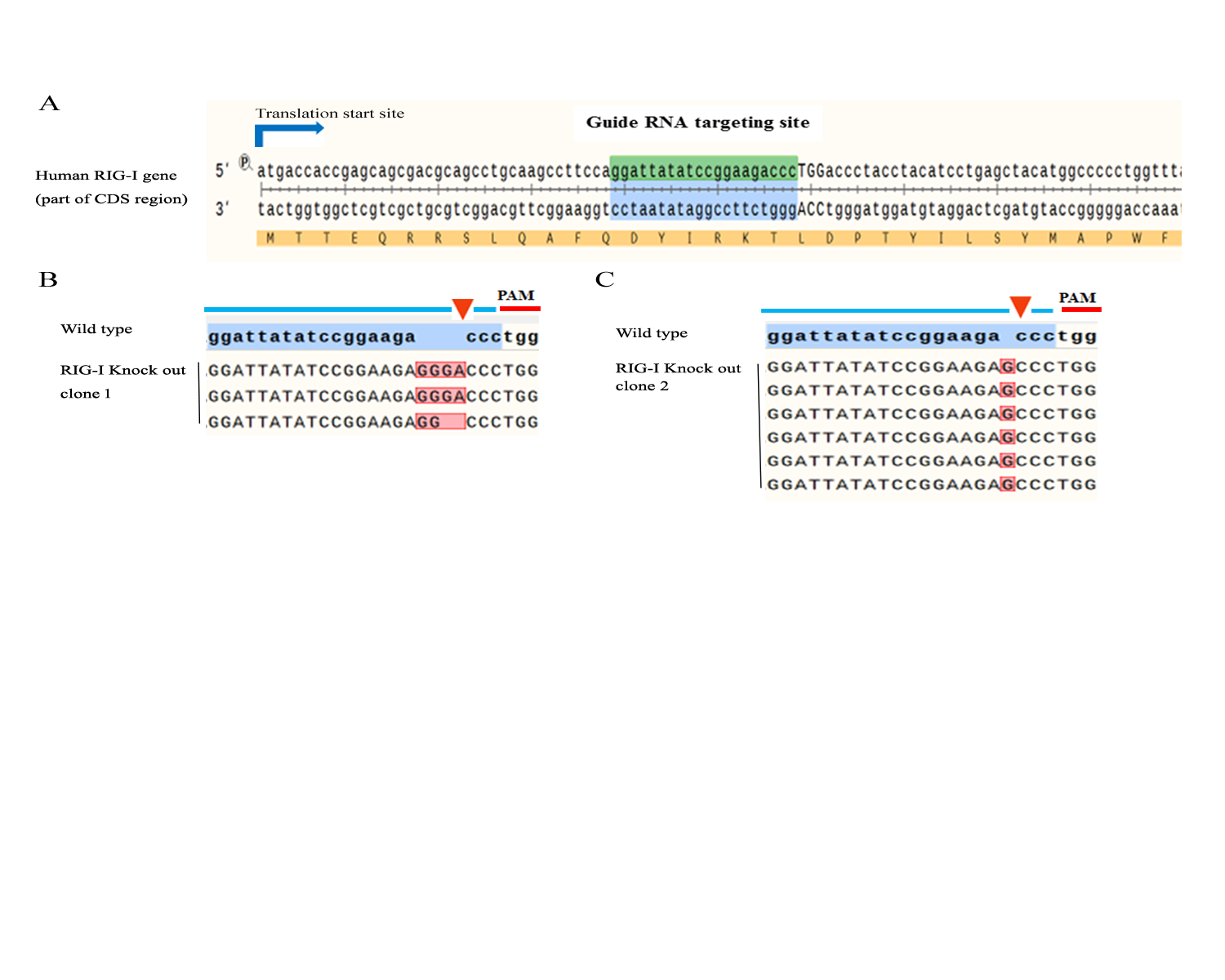

Supplement: Supplementary file 4 — Figure S4. Gene mutation information of RIG-I knockout Jurkat cell clones. (A) Illustration of the targeting site of designed guide RNA sequence, the site was 40 bases below the translation start site. (B, C) the sequencing results of Jurkat RIG-I knockout clone1 and clone 2. After the monoclones of Jurkat RIG-I knockout were obtained, the DNA region that contained the targeting site was amplicated. Then, the PCR products were cloned into T vector and sequenced by Sanger sequencing. (TIF 1270 kb) [file 13075_2019_1905_MOESM4_ESM.tif]
